# Identification of inflammatory markers suitable for non-invasive, repeated measurement studies in biobehavioral research: A feasibility study

**DOI:** 10.1371/journal.pone.0221993

**Published:** 2019-09-24

**Authors:** H. M. Schenk, S. L. van Ockenburg, M. C. Nawijn, P. De Jonge, J. G. M. Rosmalen

**Affiliations:** 1 Interdisciplinary Center Psychopathology and Emotion regulation (ICPE), University Center for Psychiatry, University of Groningen, University Medical Center Groningen, Groningen, The Netherlands; 2 Department of Pathology and Medical Biology, GRIAC Research Institute, University of Groningen, University Medical Center Groningen, Groningen, The Netherlands; Department of Psychiatry and Neuropsychology, Maastricht University Medical Center, NETHERLANDS

## Abstract

**Introduction:**

Studying the role of the immune system in the interaction between mental and physical health is challenging. To study individuals with an intensive, longitudinal study design that requires repetitive sampling in their daily life, non-invasive sampling techniques are a necessity. Urine can be collected in a non-invasive way, but this may be demanding for participants and little is known about fluctuation of inflammatory markers in urine over time. The aim of this study was to investigate the feasibility of non-invasive sampling, and to explore intra-individual differences in inflammatory markers in urine.

**Materials & methods:**

Ten healthy individuals collected 24-hour urine for 63 consecutive days. In a pilot analysis, 39 inflammatory markers were examined for detectability in urine, stability over time and under storage conditions, and daily fluctuations. Multiplex analyses were used to quantify levels of eight selected markers: C-reactive protein (CRP), Fractalkine, Interleukin-1 receptor-antagonist (IL-1RA), interferon-α (IFNα), interferon-γ (IFNγ), Interferon gamma-induced protein 10 (IP10), Macrophage inflammatory protein-1β (MIP-1β), and Vascular Endothelial Growth Factor (VEGF). Cross-correlations were calculated between the overnight and 24-hour samples were calculated, to examine whether 24-hour urine could be replaced by the overnight portion for better feasibility. We examined intra- and interindividual differences in the levels of inflammatory markers in urine and the fluctuations thereof.

**Results:**

This study showed that levels of selected inflammatory markers can be detected in urine. Cross-correlation analyses showed that correlations between levels of inflammatory markers in the night portion and the 24-hour urine sample varied widely between individuals. In addition, analyses of time series revealed striking inter- and intra-individual variation in levels of inflammatory markers and their fluctuations.

**Conclusion:**

We show that the assessment of urinary inflammatory markers is feasible in an intensive day-to-day study in healthy individuals. However, 24-hour urine cannot be replaced by an overnight portion to alleviate the protocol burden. Levels of inflammatory markers show substantial variation between and within persons.

## Introduction

The interaction between mental and physical health and the bidirectional role of the immune system therein is receiving increasing attention [1–3]. Prolonged psychological stress is associated with low-grade inflammation and an increased risk for pathology [2,4,5], while low-grade inflammation might play a role in the etiology and persistence of depression in turn [6].

Several studies have previously examined the role of low-grade inflammation in relation to stress and mental health. However, the interpretation of the results of these studies is limited for two reasons. First, studies are mostly performed in a laboratory setting thus lacking ecological validity [7]. Second, they cover only a short period of time in which the effect of one intense stressor is studied [8,9]. Preferably, the relationship between psychological factors and inflammation would be studied in an idiographic study, which focuses on the dynamics of events over time within individuals. Accordingly, an idiographic study design allows analyses at the within-person level and provides information about individual variation and the relationships between fluctuations in variables over time [10,11].

A non-clinical, everyday environment is preferable for studying psychobiological associations, representing the conditions of the real world, ameliorating the extrapolation of the outcome to a natural context [7,12,13]. To date, however, inflammatory markers are generally measured in venous blood [14]. Repeatedly collecting venous blood in an everyday environment would be far too invasive and unpractical. Fortunately, some inflammatory markers are also excreted in urine and saliva [15,16], which can be obtained in a non-invasive way. The number of markers that is measured in urine and saliva is increasing [17–20], therefore samples of these materials could be useful for ecological assessments in day-to-day idiographic studies. However, salivary assessments have their own limitations. Although non-invasive collection of saliva is possible, a major disadvantage is the necessity of extensive repeated measures per day over time, and as a consequence the high number of samples and costs. In addition, biomarkers in saliva appear to reflect mainly responses to acute stress, although several markers are also significantly or marginally increased for a longer period i.e. hours after an acute stressor [21]. The goal of this study is to investigate the feasibility of repeated measurements of inflammatory markers in urine collected and pooled over longer periods of time, and to explore inter- and intra-individual differences in urinary inflammatory markers. Before implementing non-invasive ways to measure immunological biomarkers in idiographic studies, several questions have to be addressed. The first question is which inflammatory markers are detectable and stable in urine of healthy individuals. Second, collection of all urine produced over a full 24-hour period (24-hour urine) may be challenging for participants [11]. As an alternative, the first morning void or overnight urine could be tested for biomarkers. It is unknown whether a first morning void provides sufficient insight into a person’s levels of inflammation throughout the day, and could thus replace the more burdensome 24-hour urine collection procedure. Third, idiographic studies are based on variability within individuals, thus it is important to assess to which degree levels of detectable inflammatory markers fluctuate over time. To assess if the collection of urine and measurement of inflammatory markers in urine is feasible, we set up an intensive day-to-day two-month study. We used the data generated in this study to answer the following three research questions: 1) Can inflammatory markers be assessed in urine in daily life over a prolonged period of time? 2) What is the correlation between the levels of inflammatory markers in 24-hour urine and overnight urine, which would be less burdensome to collect? 3) What is the intra-individual variability of urinary inflammatory markers?

## Materials & methods

Before conducting the current study, a pilot study was performed to test which inflammatory markers could be reliably measured in 24-hour urine of healthy individuals.

### Pilot study

It is important to realize that in a day-to-day longitudinal study, participants are engaging in their daily routines, and samples cannot be processed and stored at -80°C immediately. Therefore it was studied whether the markers were stable at room temperature for 24–48 hours. Since repeated measurement studies are meant to provide information about (bidirectional) relationships between variables over time, it was assessed whether inflammatory markers showed intra-individual variation [22].

The pilot study was performed in two parts. In part one, 24-hour urine of three healthy adults, without any current somatic and/or mental illnesses and medication use, were collected, and the presence of 39 inflammatory markers was examined: C-reactive protein (CRP), Epidermal growth factor (EGF), Eotaxin, Basic fibroblast growth factor (FGF-2), FMS-like tyrosine kinase 3 ligand (Flt-3L), Fractalkine, Granulocyte-colony stimulating factor (G-CSF), Granulocyte-macrophage colony-stimulating factor (GM-CSF), Growth regulated oncogene (GRO) chemokine, Interferon (IFN)α2, Interferon (IFN)γ, Interleukin (IL-)1RA, Interleukin (IL-)1α, Interleukin (IL-)1β, Interleukin(IL-)2, Interleukin (IL-)3, Interleukin (IL-)4, Interleukin (IL-)5, Interleukin (IL-)6, Interleukin (IL-)7, Interleukin (IL-)8, Interleukin (IL-)9, Interleukin (IL-)10, Interleukin (IL-)12p40, Interleukin (IL-)12p70, Interleukin (IL-)13, Interleukin (IL-)15, Interleukin (IL-)17A, Interferon gamma-induced protein (IP)10, Macrophage-derived chemokine (MDC), Monocyte chemoattractant protein (MCP-)1, Monocyte chemoattractant protein (MCP-)3, Macrophage inflammatory protein (MIP-)1α, Macrophage inflammatory protein (MIP-)1β, Soluble CD40 ligand (sCD40L), Transforming growth factor (TGF)α, Tumor necrosis factor (TNF)α, Tumor necrosis factor (TNF)β, and Vascular endothelial growth factor (VEGF). Furthermore, the influence of different storage conditions (room temperature, 8°C, and -20°C) on the concentration of inflammatory markers in urine samples after collection was studied using two technical replicates for the measurements.

In urine samples, 18 of the 39 tested inflammatory markers were detected: EGF, Eotaxin, Fractalkine, G-CSF, GM-CSF, IFNα2, IFNγ, IL-1RA, IL-1α, IL-7, IL-8, IL-12p70, IP-10, MDC, MCP1, sCD40L, TNFα, and VEGF. Inflammatory markers whose levels were not stable under the tested storage conditions or over time, IL-12p70, IL-17A, MDC, sCD40L, and TNF-α, were excluded.

In the second part of the pilot study, the within-individual fluctuation of the remaining inflammatory markers on a day-to-day basis was examined. We aimed to identify those inflammatory markers showing meaningful fluctuations, as defined by a day-to-day coefficient of variation (CV) of at least 10%. This resulted in the final selection of inflammatory markers of CRP, Fractalkine, G-CSF, GM-CSF, IFN-α2, IFNγ, IL-1RA, IL-7, IP10, MCP-1, MIP-1β and VEGF, that were all detectable, stable at different storage conditions, and fluctuated on a day-to-day basis.

### Main study

Based on the pilot study, eight inflammatory markers were selected for an intensive day-to-day study, namely CRP, Fractalkine, IFN-α, IFN-γ, IL-1RA, IP10, MIP-1β and VEGF.

### Subjects

The samples were initially collected for a study describing stress hormone levels in healthy individuals. This study was a longitudinal prospective observational study generating time series data of 10 healthy participants who collected 24-h urine samples for 63 consecutive days. They were paid €5,- per day of study participation, thus a total of €315,- after completion of the entire study period. A total of 11 participants were included in the study. One person discontinued participation in the study due to a major life event after two days. The protocol of the study was approved by the Medical Ethics Committee of the University Medical Center Groningen in the Netherlands (NL39630.042.12) and before enrollment all participants gave written informed consent [23]. An additional element of the informed consent included a clause in the protocol which stated that, after the study was closed in March 2012, the key to identify individuals from research data was destroyed, and all data and samples were released for further research goals. Also on this part, all participants gave written informed consent. The study described in this paper was performed after data anonymization of the participants, and therefore no written informed consent was possible or necessary anymore. Ten healthy participants (7 females) collected 24-hour urine for 63 consecutive days. Inclusion criteria were being a healthy adult between the ages of 18 and 65 years. Exclusion criteria were any current somatic and/or mental illnesses and medication use other than oral contraceptives or occasional acetaminophen. Participants were asked to daily report health complaints or use of medication by use of a web based electronic diary.

### Urine

Urine was collected in two portions. The first portion consisted of the ‘overnight portion’. Voiding during the night was also appointed to the ‘overnight portion’. The second portion consisted of the remaining voids of the day until bedtime, called the ‘day portion’. Urine containers (BD Biosciences, Franklin Lakes, NJ, USA) were weighed after collection on a scale, accurate up to 1 gram, to determine total output. The ‘day portion’ was stored at room temperature during the accumulation period. Every other morning, the researcher collected the samples and transferred them to the laboratory. Then two separate samples of the ‘overnight portion’ and ‘day portion’ were aliquoted into 2.0 ml cryotubes. Subsequently, samples were stored at -80°C until further analyses. Completeness of the 24-hour urine samples was assessed by use of 24-urinary creatinine output. In accordance with previous studies, a sample was considered incomplete if the 24 hr creatinine output was lower than 2 SD’s from the persons own mean [11,23,24], and excluded from further analyses. Samples that were higher than 2SDs from the person’s mean are not due to protocol violations but represent physiological fluctuations due to such as dietary (meat) intake or (intensive) exercise, hydration status and protein loading, and thus should not be excluded.

### Analyses of inflammatory markers

Before analysis, urine samples were centrifuged after thawing for 1650 x g at 4°C for 10 minutes. Concentration of CRP, Fractalkine, IFN-α, IFN-γ, IL-1RA, IP10, MIP-1β and VEGF in the overnight and day portion was assessed using two different magnet bead multiplex assays (Merck Millipore, Billerica, MA, USA) and a Luminex 200 analyzer (Luminex®, Austin, TX, USA), following the manufacturer’s protocol. Results were analyzed using Milliplex Analyst V5.1 software (VigeneTech Inc, Carlisle, MA, USA). Total excretion of inflammatory markers was calculated using the following equation: (concentration overnight portion (ng/ml)) * (total output overnight portion (ml)) + (concentration day portion (ng/ml)) * (total output day portion (ml)) = total excretion (ng) of inflammatory marker per day. The intra-assay and inter-assay coefficients of variance were respectively: 1.5–15% and 3.5–20%.

### Data analyses

Missing time series data was imputed using the package ‘Amelia’ [25]; the number of imputed data sets was 50. Auto Regressive Integrated Moving Average (ARIMA) models were fitted to study the association between levels of inflammatory markers as measured in the overnight and 24-hour urine portion. Each time series was detrended and demeaned, and ARIMA residuals were stored, using the package ‘astsa’ (http://www.stat.pitt.edu/stoffer/tsa4/). The value of the lag 0 of the cross-correlation function (CCF) between the ARIMA residuals of the night and 24-hour urine portion was calculated for each inflammatory marker, within each individual. Analyses were done using Rstudio (version 0.99.896, Inc., Boston, MA, http://www.rstudio.com). Descriptive statistics of eight inflammatory markers were presented in a boxplot.

## Results

### Feasibility of collection of urine in 10 healthy individuals

Urine samples of 10 different individuals (Table 1) collected on 63 consecutive days were analyzed for the concentration of the following eight inflammatory markers: CRP, Fractalkine, IFNα, IFNγ, IL-1RA, IP10, MIP-1β and VEGF. As a measure of feasibility, we assessed completeness of the 24-hour urine samples as determined by 24-hour urinary creatinine output. Based on this method the following samples were excluded for further analyses: day 23, 24, 28, 29, 32 for participant 1, day 41 for participant 2, day 63 for participant 5, day 22 for participant 6, day 1 and 34 for participant 7, day 56 for participant 8, and day 40 for participant 10. Due to technical problems, CRP results are missing for individuals 7, 8, 9 and 10.

**Table 1:** Sample characteristics.

	1	2	3	4	5	6	7	8	9	10
Sex	m	f	f	f	M	m	F	f	f	f
Age (years)	24	58	29	33	39	19	21	21	48	22
BMI	23.2	26.5	20.0	17.2	20.0	21.6	21.3	20.1	25.3	23.1
Smoking	yes	no	no	no	yes	no	no	no	no	no

### Night portion vs 24-hour urine

Cross-correlation function (CCF) was used for each inflammatory marker within each individual to assess whether overnight urine could replace the 24-hour urine. Cross-correlations between overnight urine and 24-hour urine are shown in Table 2. Cross-correlations between the overnight urine and 24-hour urine ranged from -0.053 to 0.968. IFNγ did not show enough non-zero data points to execute CCF in any of the individuals. IP10 showed significant moderate to strong correlations between overnight urine and 24-hour urine for almost all individuals. ID 4, 5, 8 and 10 showed significant correlations for all inflammatory markers between overnight urine and 24-hour urine.

### Inter- and intra-individual differences

**Fig 1:**
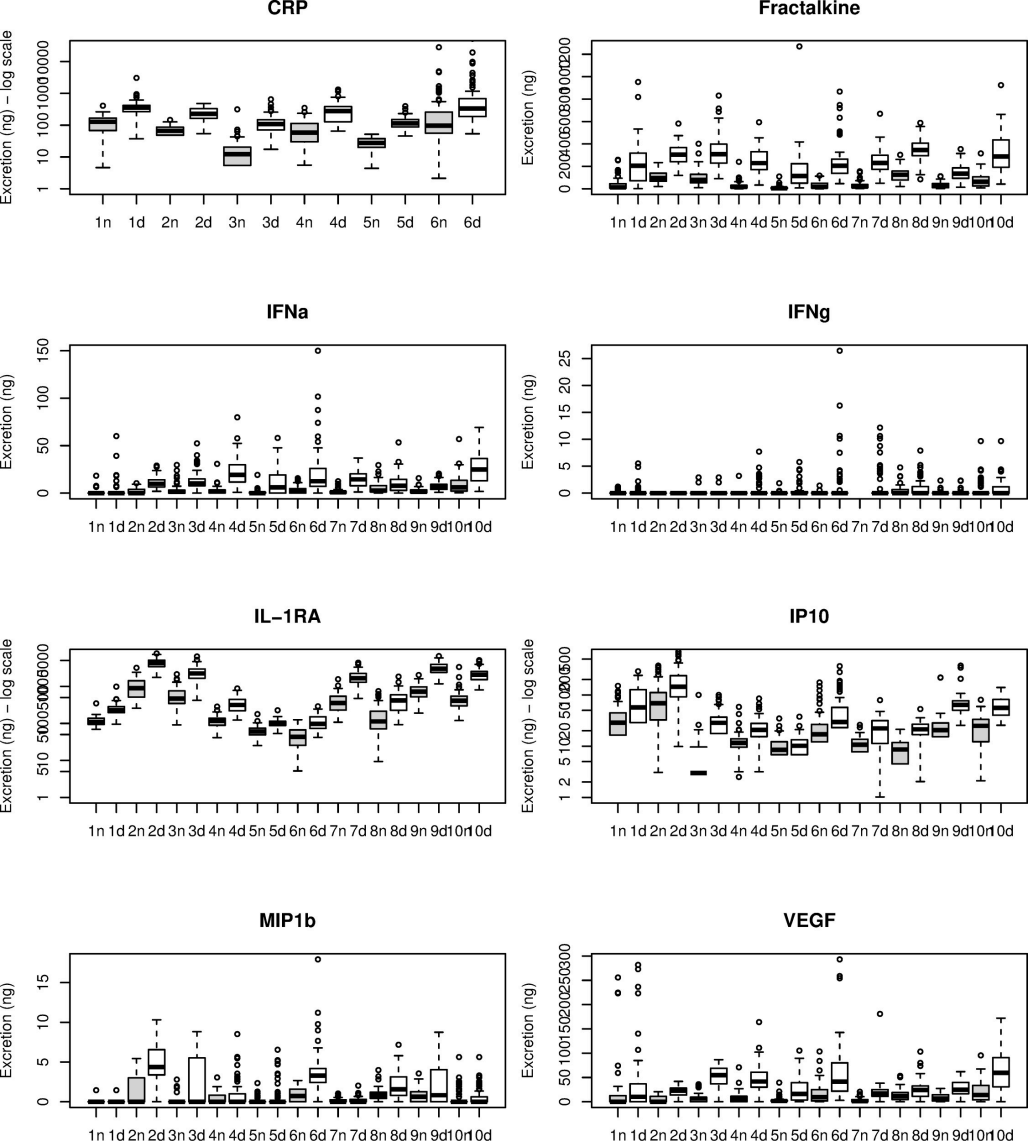
Boxplot showing inter- and intra-individual differences of eight inflammatory markers, measured in overnight and day urine, collected during 63 consecutive days in an idiographic study, showing median and interquartile levels of each participant. CRP = C-reactive protein, IFN = Interferon, IL-1RA = Interleukin-1 receptor antagonist, IP10 = Interferon gamma-induced protein 10, MIP1β = Macrophage Inflammatory Proteins, VEGF = Vascular endothelial growth factor, ng = nanogram, the number on the x-axis represents the individual, n = overnight (gray columns), d = 24-hour urine (white colums).

Each inflammatory marker showed considerable differences in median levels and interquartile ranges (IQR) between and within different participants (Fig 1). ID 5 showed overall the lowest median excretion of inflammatory markers (Fig 1, white columns). ID 2 and ID 10 showed the highest median excretion of inflammatory markers (Fig 1, white columns). The differences between the excretion levels are substantial in several cases, e.g. the interquartile ranges of CRP in ID 1 and 3, and ID 5 and 6 do not overlap and median levels show a difference of around 100% in those participants. The same holds true for levels of Fractalkine in ID 8 and ID 9, or IFNα in ID 9 and ID 10. Or even more prominent, ranges between IL-1RA excretion levels in ID1 and ID2, or ID 2 and ID 5 show great differences.

**Table 2:** Correlation between excretion of inflammatory markers in overnight and 24-hour urine.

ID	CRP	Fractalkine	IFNα	IFNγ	IL-1RA	IP10	MIP1β	VEGF
1	0.161	0.341^a^	0.178	NA	0.490^a^	0.197	NA	0.747^a^
2	0.559^a^	0.048	0.229	NA	0.556^a^	0.738^a^	0.458^a^	0.330^a^
3	0.087	0.034	-0.053	NA	0.138	0.465^a^	0.156	0.245
4	0.372^a^	0.423^a^	0.528^a^	NA	0.321^a^	0.621^a^	0.636^a^	0.528^a^
5	0.373^a^	0.410^a^	0.532^a^	NA	0.330^a^	0.610^a^	NA	0.530^a^
6	0.968^a^	0.028	0.008	NA	0.625^a^	0.691^a^	0.342^a^	0.428^a^
7	NA	0.409^a^	0.234	NA	0.360^a^	0.528^a^	NA	0.094
8	NA	0.508^a^	0.475^a^	NA	0.524^a^	0.422^a^	0.411^a^	0.448^a^
9	NA	0.400^a^	0.532^a^	NA	-0.036	0.785^a^	0.307^a^	0.510^a^
10	NA	0.296^a^	0.594^a^	NA	0.463^a^	0.577^a^	NA	0.464^a^

Results of the cross-correlation function (CCF), analyzing the correlation (*B*) between the overnight portion and 24-hour urine. N = 63 for each correlation. IFNγ did not show enough non-zero data points to execute CCF. Due to technical issues regarding the assays, no results for CRP were acquired for individual 7, 8, 9 and 10. Number of imputed data sets was 50. Each time series was detrended and demeaned before analysis.

^a^ indicates values that surpass 95% confidence intervals, indicating a significant correlation (p ≤ 0.05), 95% CI = 0.252 (±2/√*n*). NA = not applicable.

## Discussion

In this study we showed that several inflammatory markers can be assessed in urine in daily life over a prolonged period of time. Our results also revealed that 24-hour urine cannot be replaced by overnight urine, since there was only a moderate correlation between levels of inflammatory markers in overnight urine and 24-hour urine. Furthermore, explorative analyses showed high inter-and intra-individual variability of urinary inflammatory markers over time.

Day-to-day studies require non-invasive techniques to diminish the burden of the study on participants. Urine is appealing for research in the field of behavioral science, since it is a direct filtrate of the blood and can be collected using non-invasive techniques. Moreover, urine contains an accumulation of small metabolites which can cross the glomerular filtration barrier, thus providing an integrative measure of low grade inflammation. Based on the literature and a pilot study, we selected eight inflammatory markers to be measured in a repeated measurement studies. The biomarkers that we selected are in line with literature in the field of biopsychology [15,26,27] A drawback of urinary markers is that their levels are influenced by health status, since the kidney produces or excretes many molecules as a response to inflammatory disease or kidney injury [28] Knowledge about health conditions of participants, or the presence of disease, symptoms, injuries or use of medication affecting the body, kidney or urinary tract during the study period, is therefore crucial.

The associations between overnight urine and 24-hour urine range from ‘very weak and negative’ to ‘very strong and positive’ (Table 2). Although the majority of the correlations were significant, the correlations between the overnight urine and 24-hour urine showed a wide range for different markers within each individual, and also a wide range between individuals. Due to this variety, overnight urine is not a suitable alternative for 24-hour urine. The sometimes large differences, and thus low correlation, between excretion of inflammatory markers in overnight urine and 24-hour urine are partly explained by the fact that 24-hour urine portion is larger and covers a wider time period than the overnight portion. Also, urine production during the night is decreased, which might lead to a higher concentration, but overall a lower excretion. Explorative comparison of the concentration and the total excretion of the inflammatory markers in overnight urine and the urine of the successive day, showed that the concentration of inflammatory markers (ng/ml) in the overnight urine was often higher compared to that in the urine collected during the day. Total excretion of inflammatory markers on the other hand, was in general higher during the day (data not shown). Literature shows when following a normal day-night rhythm, it appears that during sleep a shift occurs from an “inflammatory” to an “anti-inflammatory” state. [29,30] Physiological processes such as the circadian rhythm and sleep duration or quality might influence expression of certain inflammatory markers [31,32]. Disrupting the circadian rhythm, e.g. during night shifts, stimulates inflammatory activity and is associated with various adverse health effects. [33,34]

Several studies already showed that cytokine patterns are highly heterogeneous between individuals [15,35–37], however, no study showed that this heterogeneity is also present within individuals. The heterogeneity in levels of inflammatory markers is striking. Heterogeneity in the levels of inflammatory markers within individuals might be due to stress, affect, behavior or life style. In addition, visual inspection raises the questions whether time series of inflammatory markers can be translated to immunological profiles. It may be speculated that such profiles might reflect specific susceptibilities and have relevance for health outcomes.

The strength of this study is its novelty due to the assessment of eight inflammatory markers in a repeated measurement study design. The idiographic study design gives insight into the large differences between individuals and the fluctuation of the inflammatory markers within individuals, and underlines this often ignored limitation of outcomes of group studies. This study has limitations that need to be mentioned as well. Although urine is often used for detection of specific proteins, the mentioned arrays were not specifically tested for the use of urine, as a consequence cross-reactivity and influence of e.g. pH was not completely ruled out. The high costs of the analyses techniques forced us to narrow down the number of inflammatory markers in the cohort study.

In conclusion, we showed that the measurement of inflammatory markers in urine has the potential for successful incorporation in idiographic research. The data also confirms the need for a longitudinal approach in biobehavioral research, since cross-sectional data would not have uncovered the wide distribution of inflammation levels within-individuals. In future research, intensive day-to-day studies might provide new insights into the role of low-grade inflammation in individual psychological processes.
